# Supplementary material for: Pex30-like proteins function as adaptors at distinct ER membrane contact sites
Source: J Cell Biol. 2021 Aug 17;220(10):e202103176. doi: 10.1083/jcb.202103176 (PMC8374871; doi:10.1083/jcb.202103176)
Supplement: Table S1 — lists the yeast strains used in this study. [file JCB_202103176_TableS1.docx]

**Table S1. Yeast strains used in this study**

| **Strain** | **Genotype** |
| --- | --- |
| BY4741 | *Mat a ura3∆0 HIS33∆1 leu2∆0 met15∆0* |
| yPC3975 | *Mat a ura3∆0 HIS33∆1 leu2∆0 met15∆0 sei1∆::KAN* |
| yPC8806 | *Mat a ura3∆0 HIS33∆1 leu2∆0 met15∆0 pex28∆::KAN* |
| yPC8807 | *Mat a ura3∆0 HIS33∆1 leu2∆0 met15∆0 pex29∆::KAN* |
| yPC8808 | *Mat a ura3∆0 HIS33∆1 leu2∆0 met15∆0 pex30∆::KAN* |
| yPC8809 | *Mat a ura3∆0 HIS33∆1 leu2∆0 met15∆0 pex31∆::KAN* |
| yPC8810 | *Mat a ura3∆0 HIS33∆1 leu2∆0 met15∆0 pex32∆::KAN* |
| yPC8845 | *Mat a ura3∆0 HIS33∆1 leu2∆0 met15∆0 pex28∆::KAN sei1∆::NAT* |
| yPC8848 | *Mat a ura3∆0 HIS33∆1 leu2∆0 met15∆0 pex29∆::KAN sei1∆::NAT* |
| yPC8849 | *Mat a ura3∆0 HIS33∆1 leu2∆0 met15∆0 pex30∆::KAN sei1∆::NAT* |
| yPC8855 | *Mat a ura3∆0 HIS33∆1 leu2∆0 met15∆0 pex31∆::KAN sei1∆::NAT* |
| yPC8858 | *Mat a ura3∆0 HIS33∆1 leu2∆0 met15∆0 pex32∆::KAN sei1∆::NAT* |
| yPC10657 | *Mat a ura3∆0 HIS33∆1 leu2∆0 met15∆0* Pex30-mNeonGreen::HIS3 |
| yPC10676 | *Mat a ura3∆0 HIS33∆1 leu2∆0 met15∆0* Pex30-mNeonGreen::HIS3 *sei1::NAT* |
| yPC10782 | *Mat a ura3∆0 HIS33∆1 leu2∆0 met15∆0* Pex30-tdTomato::HIS3 |
| yPC10783 | *Mat a ura3∆0 HIS33∆1 leu2∆0 met15∆0* Pex30-tdTomato::HIS3 *sei1∆::KAN* |
| yPC10865 | *Mat a ura3∆0 HIS33∆1 leu2∆0 met15∆0* Pex30-mNeonGreen::KAN Nvj1-tdTomato::HIS3 |
| yPC11183 | *Mat a ura3∆0 HIS33∆1 leu2∆0 met15∆0* Pex30-mNeonGreen::HIS3 *pex29∆::KAN* |
| yPC11184 | *Mat a ura3∆0 HIS33∆1 leu2∆0 met15∆0* Pex30-mNeonGreen::HIS3 *pex31∆::KAN* |
| yPC11185 | *Mat a ura3∆0 HIS33∆1 leu2∆0 met15∆0* Pex30-mNeonGreen::HIS3 *pex32∆::KAN* |
| yPC11211 | *Mat a ura3∆0 HIS33∆1 leu2∆0 met15∆0* Pex30-mNeonGreen::HIS3 *pex28∆::KAN* |
| yPC11360 | *Mat a ura3∆0 HIS33∆1 leu2∆0 met15∆0* Pex30*(Δ284-408)*-mNeonGreen::HIS3*(CRISPR)* |
| yPC11363 | *Mat a ura3∆0 HIS33∆1 leu2∆0 met15∆0* Pex30*(Δ284-408)*-tdTomato::HIS3*(CRISPR)* |
| yPC11364 | *Mat a ura3∆0 HIS33∆1 leu2∆0 met15∆0* Pex30*(Δ284-408)*-tdTomato::HIS3*(CRISPR) sei1∆::KAN* |
| yPC11419 | *Mat a ura3∆0 HIS33∆1 leu2∆0 met15∆0 NAT::GPDp-3xHA-Pex28 pex30∆::KAN* |
| yPC11420 | *Mat a ura3∆0 HIS33∆1 leu2∆0 met15∆0 NAT::GPDp-3xHA-Pex29 pex30∆::KAN* |
| yPC11421 | *Mat a ura3∆0 HIS33∆1 leu2∆0 met15∆0 NAT::GPDp-3xHA-Pex31 pex30∆::KAN* |
| yPC11423 | *Mat ? ura3∆0 HIS33∆1 leu2∆0 met15∆0 NAT::GPDp-3xHA-Pex28* |
| yPC11427 | *Mat ? ura3∆0 HIS33∆1 leu2∆0 met15∆0 NAT::GPDp-3xHA-Pex28 pex30∆::KAN sei1∆::HIS3* |
| yPC11430 | *Mat ? ura3∆0 HIS33∆1 leu2∆0 met15∆0 NAT::GPDp-3xHA-Pex29* |
| yPC11433 | *Mat ? ura3∆0 HIS33∆1 leu2∆0 met15∆0 NAT::GPDp-3xHA-Pex29 pex30∆::KAN sei1∆::HIS3* |
| yPC11436 | *Mat a ura3∆0 HIS33∆1 leu2∆0 met15∆0 NAT::GPDp-3xHA-Pex31* |
| yPC11440 | *Mat ? ura3∆0 HIS33∆1 leu2∆0 met15∆0 NAT::GPDp-3xHA-Pex31 pex30∆::KAN sei1∆::(CRISPR)* |
| yPC11490 | *Mat a ura3∆0 HIS33∆1 leu2∆0 met15∆0* Pex28-3xHA::KAN |
| yPC11491 | *Mat a ura3∆0 HIS33∆1 leu2∆0 met15∆0* Pex29-3xHA::KAN |
| yPC11492 | *Mat a ura3∆0 HIS33∆1 leu2∆0 met15∆0* Pex30-3xHA::KAN |
| yPC11493 | *Mat a ura3∆0 HIS33∆1 leu2∆0 met15∆0* Pex31-3xHA::KAN |
| yPC11494 | *Mat a ura3∆0 HIS33∆1 leu2∆0 met15∆0* Pex32-3xHA::KAN |
| yPC11544 | *Mat a ura3∆0 HIS33∆1 leu2∆0 met15∆0* Pex28-3xHA::KAN *pex30∆::(CRISPR)* |
| yPC11545 | *Mat a ura3∆0 HIS33∆1 leu2∆0 met15∆0* Pex29-3xHA::KAN *pex30∆::(CRISPR)* |
| yPC11546 | *Mat a ura3∆0 HIS33∆1 leu2∆0 met15∆0* Pex31-3xHA::KAN *pex30∆::(CRISPR)* |
| yPC11547 | *Mat a ura3∆0 HIS33∆1 leu2∆0 met15∆0* Pex32-3xHA::KAN *pex30∆::(CRISPR)* |
| yPC11550 | *Mat a ura3∆0 HIS33∆1 leu2∆0 met15∆0* Pex31-mNeonGreen::KAN |
| yPC11797 | *Mat a ura3∆0 HIS33∆1 leu2∆0 met15∆0* Pex28-mNeonGreen::HYGB mCherry-PTS1::LEU2*(HO locus)* |
| yPC11798 | *Mat a ura3∆0 HIS33∆1 leu2∆0 met15∆0* Pex29-mNeonGreen::HYGB mCherry-PTS1::LEU2*(HO locus)* |
| yPC11799 | *Mat a ura3∆0 HIS33∆1 leu2∆0 met15∆0* Pex30-mNeonGreen::HIS3 mCherry-PTS1::LEU2*(HO locus)* |
| yPC11801 | *Mat a ura3∆0 HIS33∆1 leu2∆0 met15∆0* Pex32-mNeonGreen::HYGB mCherry-PTS1::LEU2*(HO locus)* |
| yPC11803 | *Mat a ura3∆0 HIS33∆1 leu2∆0 met15∆0 pex30Δ::KAN* mCherry-PTS1::LEU2*(HO locus)* |
| yPC11829 | *Mat a ura3∆0 HIS33∆1 leu2∆0 met15∆0* Pex28-3xHA::KAN *pex31∆::(CRISPR)* |
| yPC11830 | *Mat a ura3∆0 HIS33∆1 leu2∆0 met15∆0* Pex29-3xHA::KAN *pex31∆::(CRISPR)* |
| yPC11832 | *Mat a ura3∆0 HIS33∆1 leu2∆0 met15∆0* Pex32-3xHA::KAN *pex31∆::(CRISPR)* |
| yPC11837 | *Mat ? ura3∆0 HIS33∆1 leu2∆0 met15∆0* Pex28-mNeonGreen::HYGB Nvj1-tdTomato::HIS3 |
| yPC11840 | *Mat ? ura3∆0 HIS33∆1 leu2∆0 met15∆0* Pex29-mNeonGreen::HYGB Nvj1-tdTomato::HIS3 |
| yPC11843 | *Mat ? ura3∆0 HIS33∆1 leu2∆0 met15∆0* Pex30-mNeonGreen::HYGB Nvj1-tdTomato::HIS3 |
| yPC11846 | *Mat ? ura3∆0 HIS33∆1 leu2∆0 met15∆0* Pex31-mNeonGreen::HYGB Nvj1-tdTomato::HIS3 |
| yPC11849 | *Mat ? ura3∆0 HIS33∆1 leu2∆0 met15∆0* Pex32-mNeonGreen::HYGB Nvj1-tdTomato::HIS3 |
| yPC11876 | *Mat a ura3∆0 HIS33∆1 leu2∆0 met15∆0* Pex28-13xMyc::HIS3 Pex29-V5::(CRISPR) Pex32-3xHA::KAN |
| yPC11878 | *Mat a ura3∆0 HIS33∆1 leu2∆0 met15∆0* Pex28-13xMyc::HIS3 Pex32-3xHA::KAN *pex29∆::(CRISPR)* |
| yPC11879 | *Mat a ura3∆0 HIS33∆1 leu2∆0 met15∆0* Pex29-V5::(CRISPR) Pex32-3xHA::KAN *pex28∆::(CRISPR)* |
| yPC11880 | *Mat a ura3∆0 HIS33∆1 leu2∆0 met15∆0* Pex28-13xMyc::HIS3 Pex29-V5::(CRISPR) *pex32∆::(CRISPR)* |
| yPC11898 | *Mat ? ura3∆0 HIS33∆1 leu2∆0 met15∆0* Pex30*(Δ410-523)*-tdTomato::HIS3 *sei1∆::KAN* |
| yPC11899 | *Mat a ura3∆0 HIS33∆1 leu2∆0 met15∆0* Pex30*(Δ410-523)*-tdTomato::HIS3 |
| yPC11971 | *Mat a ura3∆0 HIS33∆1 leu2∆0 met15∆0* Pex28-13xMyc::HIS3 Pex29-V5::(CRISPR) Pex32-3xHA::KAN Pex30*(2-59∆)::(CRISPR)* |
| yPC11975 | *Mat a ura3∆0 HIS33∆1 leu2∆0 met15∆0* Pex28-13xMyc::HIS3 Pex29-V5::(CRISPR) Pex32-3xHA::KAN Pex30*(60-160∆)::(CRISPR)* |
| yPC11976 | *Mat a ura3∆0 HIS33∆1 leu2∆0 met15∆0* Pex28-13xMyc::HIS3 Pex29-V5::(CRISPR) Pex32-3xHA::KAN Pex30*(284-408∆)::(CRISPR)* |
| yPC11983 | *Mat a ura3∆0 HIS33∆1 leu2∆0 met15∆0* Pex30-mNeonGreen::HIS3 *pex28∆::KAN* mCherry-PTS1::LEU2*(HO Locus)* |
| yPC11984 | *Mat a ura3∆0 HIS33∆1 leu2∆0 met15∆0* Pex30-mNeonGreen::HIS3 *pex29∆::KAN* mCherry-PTS1::LEU2*(HO Locus)* |
| yPC11985 | *Mat a ura3∆0 HIS33∆1 leu2∆0 met15∆0* Pex30-mNeonGreen::HIS3 *pex31∆::KAN* mCherry-PTS1::LEU2*(HO Locus)* |
| yPC11986 | *Mat a ura3∆0 HIS33∆1 leu2∆0 met15∆0* Pex30-mNeonGreen::HIS3 *pex32∆::KAN* mCherry-PTS1::LEU2*(HO Locus)* |
| yPC12051 | *Mat a ura3∆0 HIS33∆1 leu2∆0 met15∆0* Pex30*(Δ284-408)*-mNeonGreen::HIS3(*CRISPR)* mCherry-PTS1::LEU2*(HO locus)* |
| yPC12056 | *Mat a ura3∆0 HIS33∆1 leu2∆0 met15∆0* Pex28-13xMyc::HIS3 Pex29-V5::(CRISPR) Pex32-3xHA::KAN *pex31∆::(CRISPR)* |
| yPC12063 | *Mat a ura3∆0 HIS33∆1 leu2∆0 met15∆0* Pex30-mNeonGreen::KAN Nvj1-tdTomato::HIS3 *pex29∆::(CRISPR)* |
| yPC12104 | *Mat a ura3∆0 HIS33∆1 leu2∆0 met15∆0* Pex28-13xMyc::HIS3 Pex29-V5::(CRISPR) Pex32-3xHA::KAN Pex30*(161-190∆)::(CRISPR)* |
| yPC12107 | *Mat a ura3∆0 HIS33∆1 leu2∆0 met15∆0* Pex28-13xMyc::HIS3 Pex29-V5::(CRISPR) Pex32-3xHA::KAN *pex30∆::(CRISPR)* |
| yPC12126 | *Mat a ura3∆0 HIS33∆1 leu2∆0 met15∆0* Pex28-13xMyc::HIS3 Pex29-V5::(CRISPR) Pex32-3xHA::KAN Pex30*(86-219∆+Rtn1(18-160))::(CRISPR)* |
| yPC12128 | *Mat a ura3∆0 HIS33∆1 leu2∆0 met15∆0* Pex28-13xMyc::HIS3 Pex29-V5::(CRISPR) Pex32-3xHA::KAN Pex31*(78-211∆ + Pex30(86-219))::(CRISPR) pex30∆::HYGB* |
| yPC12142 | *Mat ? ura3∆0 HIS33∆1 leu2∆0 met15∆0 pex28∆::KAN pex29∆::HYGB pex31∆::NAT pex32∆::KAN* |
| yPC12148 | *Mat a ura3∆0 HIS33∆1 leu2∆0 met15∆0* Pex28-13xMyc::HIS3 Pex29-V5::(CRISPR) Pex32-3xHA::KAN Pex30*(415-513∆)::(CRISPR)* |
| yPC12150 | *Mat ? ura3∆0 HIS33∆1 leu2∆0 met15∆0 pex28∆::KAN pex29∆::HYGB pex31∆::NAT pex32∆::KAN sei1::NAT* |
| yPC12175 | *Mat ? ura3∆0 HIS33∆1 leu2∆0 met15∆0* Pex30-mNeonGreen::HIS3 *pex28∆::KAN pex29∆::HYGB pex31∆::NAT pex32∆::KAN* |
| yPC12176 | *Mat ? ura3∆0 HIS33∆1 leu2∆0 met15∆0* Pex30-mNeonGreen::HIS3 *pex28∆::KAN pex29∆::HYGB pex31∆::NAT pex32∆::KAN sei1∆::NAT* |
| yPC12183 | *Mat a ura3∆0 HIS33∆1 leu2∆0 met15∆0* Pex28-13xMyc::HIS3 Pex29-V5::(CRISPR) Pex32-3xHA::KAN Pex30*(86-219∆+Pex31(78-211))::(CRISPR)* |
| yPC12185 | *Mat a ura3∆0 HIS33∆1 leu2∆0 met15∆0* Pex28-13xMyc::HIS3 Pex29-V5::(CRISPR) Pex32-3xHA::KAN Pex31*(78-211∆ + Pex30(86-219))*-mNeonGreen::HYGB *pex30∆::ZEO* |
| yPC12192 | *Mat a ura3∆0 HIS33∆1 leu2∆0 met15∆0* Pex28-13xMyc::HIS3 Pex29-V5::(CRISPR) Pex32-3xHA::KAN Pex31*(78-211∆ + Pex30(86-219))::(CRISPR) pex30∆::HYGB* mCherry-PTS1::LEU2*(HO Locus)* |
| yPC12265 | *Mat a ura3∆0 HIS33∆1 leu2∆0 met15∆0* Pex28-13xMyc::HIS3 Pex29-V5::(CRISPR) Pex32-3xHA::KAN Pex30-Flag::HYGB |
| yPC12266 | *Mat a ura3∆0 HIS33∆1 leu2∆0 met15∆0* Pex28-13xMyc::HIS3 Pex29-V5::(CRISPR) Pex32-3xHA::KAN Pex30(60-283)-Flag::HYGB |
| yPC12270 | *Mat a ura3∆0 HIS33∆1 leu2∆0 met15∆0* Pex28-13xMyc::HIS3 Pex29-V5::(CRISPR) Pex32-3xHA::URA3 Pex31*(78-211∆ + Pex30(86-219))*-mNeonGreen::HYGB *pex30∆::ZEO* Nvj1-tdTomato::KAN |
| yPC12287 | *Mat a ura3∆0 HIS33∆1 leu2∆0 met15∆0* Pex30*(Δ284-408)*-mNeonGreen::HIS3*(CRISPR)* Nvj1-tdTomato::KAN |
