# Supplementary material for: Pex30-like proteins function as adaptors at distinct ER membrane contact sites
Source: J Cell Biol. 2021 Aug 17;220(10):e202103176. doi: 10.1083/jcb.202103176 (PMC8374871; doi:10.1083/jcb.202103176)
Supplement: Table S2 — lists the plasmids used in this study. [file JCB_202103176_TableS2.docx]

**Table S2. Plasmids used in this study**

| **Plasmid** | **Recombinant DNA** |
| --- | --- |
| pPC220 | pFA6a-3xHA-KANMX6 |
| pPC225 | pFA6a-13xMyc-HIS3MX6 |
| pPC655 | pFA6a-tdTomato-HIS3MX6 |
| pPC930 | pFA6a-natNT2-GPD-3xHA |
| pPC1463 | ADH1p-PTS1-mCherry |
| pPC1517 | pFA6a-mNeonGreen-HIS3MX6 |
| pPC1738 | pML107-Pex30-gRNA1 (GATGGGTCGACAAGACATGG) |
| pPC1740 | pML107-Pex30-gRNA3 (AACACCGCCCACAATCTCAA) |
| pPC1752 | pML107-Pex28-gRNA2 (TTGGAGAAACCCTACCGGGA) |
| pPC1755 | pML107-Pex29-gRNA2 (CTAGTTTGAGAATATACCAG) |
| pPC1757 | pML107-Pex31-gRNA1 (AGAAGTGTTTACTTTACCAG) |
| pPC1761 | pML107-Pex32-gRNA2 (GATAGCTTCGTTAAGAGACA) |
| pPC1854 | pML107-Pex29-gRNA4 (TAATCTCAACAATATCGACG) |
| pPC1871 | pML107-Pex31-gRNA5 (TGTTAGAAAAATATAAACTG) |
| pPC1873 | pWS176 - ZeoR |
| pPC2055 | pML107-Pex30-gRNA10 (TTGCAGCGTCTCTGCAAACT) |
| pPC2056 | pML107-Pex30-gRNA11 (ACGCTGCAACCGAGGATTGG) |
| pPC2057 | pML107-Pex30-gRNA12 (AGCTGAAAGCGGGGTGTCTG) |
| pPC2058 | pML107-Pex30-gRNA13 (GGATAATGATAACGGGTCAT) |
| pPC2059 | pML107-Pex30-gRNA14 (CGTTCCAAGGTTAACCATGG) |
| pPC2060 | pML107-Pex30-gRNA15 (TAAAACTTCTTCATCGGTCG) |
