# Supplementary material for: Pex30-like proteins function as adaptors at distinct ER membrane contact sites
Source: J Cell Biol. 2021 Aug 17;220(10):e202103176. doi: 10.1083/jcb.202103176 (PMC8374871; doi:10.1083/jcb.202103176)
Supplement: Table S3 — lists the oligonucleotides used in this study. [file JCB_202103176_TableS3.docx]

**Table S3. Oligonucleotides used in this study.**

| **Primer** | **Used on** | **Nucleotide sequence** |
| --- | --- | --- |
| 3824 | Chimeric Pex30 with Rtn1 RHD (forward) | TGACATCAACACCGCCCACAATCTCAAAAGCGTTGGTGAAATTGTACCCTTATTTAATTCTCATTAGCTGTAATTGCGATTTGCTATTAT |
| 3825 | Chimeric Pex30 with Rtn1 RHD (reverse) | ACAAGCAGCCTGACAATCTTAAACTTCCATAAAAGCCTTCTTGCTACTTTAGACCATGGTGAGTGCAAAGTGAAGGTAAAAATGTCAGCA |
| 3915 | Deletion of Sei1 ORF | TATAAATGCGCGTAAGACAGAAAAATAGAGACAGCTTACGGCAAATTATCAAAATGTGAATCCAAGGTTTCAAGAAAATAAGATAAAGTGAATAGGAAGGTAGAATTGTACTTCTCGCTATATAATTTTAAAACCTAGCTGTTATTTTCTAAGTAAGTAGGCTCTTCCAGCATTCTGCTTCTTCGCCCTGAATAAAAAAA |
| 3917 | Deletion of Pex30 ORF | CTTGGATACGTTCGCTACTGAGGTAAAGTGGGCATGCAAGGTTGCAATTTAGGATTCGAGCTGTCTAGTTGATCCTCCGGAGTGTAAAAACTGATTTTCATGCTCGCTCCCGTTTTTACCTTTACATAATATAATCTCTAATTTACTTTATTACACAAATGGTAACTCCCTTTAAATAAACTTAAAAAAGAACGTATAGA |
| 3921 | Deletion of Pex28 ORF | CGAATGCCTACGAATTGGAGTCATCAGTAGATAGAACCATAATGAATAAGATAAAAACGCAGGTATTTAGGGTGACAAAGAGATAATATATCTTCAGCAATGCACAATTGTTTTCCTTCACCTACAACCCCACCCCTCTTCTTGGGGAAATCATTGTATTACGTACTTTTTTCCTAAAGATATTAGAAAAAAAAATGTTA |
| 3922 | Deletion of Pex29 ORF | ACGAGGAATAGTGTGTATTTTTAATTACTGATCAGGCCGCATGAATATACTCACCTGTATACGTAGCAAAATTTCAGTCTCCTACTGTATATACGTCTAGACTTGATTTGTTATACTATATGTTCACTGATGATACAGTCAACTTTTCATAAAACTTTCTAATGACAGTGACGAACTTTTCTTCTTTTCCATTTTTTTCT |
| 3923 | Deletion of Pex31 ORF | TATAAGAGAAGCTCACCAAAATTATTATCAATAAAGCGGCACTTTTAAATTTGGTATTCTTCCCTGGTTGTCAAGCCTTGGTTTCCCTTTATTTGATAGTTGAGTGCATGCCCCATATGGACAACAACGTTCACACTGGTCGGTGCAAGGGCACACAGCAATCTCAGAGTTTCTTTCATTGGCTGCATGGAGCACAAATT |
| 3924 | Deletion of Pex32 ORF | AGAAGGTGCAATCACTCGAAACTACTTCATACTTTCTCTTTTCTTTCATTGATCTTAATTTAGTTCAGTGGACATCATTTTGCTAATTTCAAGGAAAGAATAGTATTGCTTTAAGCCTCTAAACTGCATATATAGTTGGTTGCCTAACATTATCATGATATGAAGAATTTTCTCATACTTTGAAACATAGGACAAGTAAA |
| 3926 | Deletion of Pex30 residues 60-160 | TACTACAAAAGTGATACGAGCTGCTTTGGAAAAGAACGAAGCTGAAAGCGGGGTGTCTGAGGATAATGATAACGGGTCATTGGAGAAAGTTAACGTAGCTAGAGTATCCTTGAAGTCAGATATTTTGCTCTCCCCCATGGTTAACCTTGGAACGCAAGATATTCAACGGCTTCTATATACTACGGTCATATTATCTCCAA |
| 3927 | Deletion of Pex30 residues 284-408 | TGGAATAAATAAGGACCAGGGTATTTTTGCTACAGTGCAGAAGCAAGTGAAAAAGTTGGCATCAACAGAAAACAGTAATGGCGTATTATCCGATTCCAAGAAAACTTCTGACTTTGATGAAAGCGTGATAAATTCGAATAGAAATTCTGCCATTGAACAAAAGGTTGAAGAAAACAGCACGAATGGTTTAACCGCTGAGC |
| 3948 | C-terminal insertion of V5 tag on Pex29 ORF | CAACAATATCGACGCCGATGCATCTTATCCGTCAATCGAAGAGCTAACAGACACTCTCAATTCAACTATAGGGGGAGGCGGGGGTGGAAAGCCTATCCCTAACCCTCTCCTCGGTCTCGATTCTACGTAGACTTGATTTGTTATACTATATGTTCACTGATGATACAGTCAACTTTTCATAAAACTTTCTAATGACAGTG |
| 4069 | Deletion of Pex30 residues 415-513 | TACCTGGAAAAAACCAAGTAAAGAAGATTCCTTTTCTAAATATACAAGAAGAAGAAGATGGGTAAGAACCGCAGAATTGGTCAAAACTTCTGACTTTGATACCATTGGTCGCGATAGCAAGAAGGCCGTATGATGCTCGCTCCCGTTTTTACCTTTACATAATATAATCTCTAATTTACTTTATTACACAAATGGTAACT |
| 4072 | Chimeric Pex30 with Pex31 RHD (forward) | AAAGGCGTTGGTGAAATTGTACCCTTATTTAATTCTCATTGATAACTTTCTAAGTATCAT |
| 4073 | Chimeric Pex30 with Pex31 RHD (reverse) | CCATAAAAGCCTTCTTGCTACTTTAGACCATGGTGAGTGGTACGTAAGCACATACAGAC |
| 4074 | Chimeric Pex31 with Pex30 RHD (forward) | CAGGTCATTAGTGAGGTTATACCCATATTTAATTGTTGTGGACGAATTCCTAAACGTTGT |
| 4075 | Chimeric Pex30 with Pex31 RHD (forward) | CCACAAGTATCTTCTCATTCTTCGGATTAGTTTAGAATGATACGTTAAAAGGAACATGC |
